# Practical Notes on Diagnosis and Treatment

**Published:** 1907-09-21

**Authors:** 


### Practical Notes on Diagnosis and Treatment.

**Hæmoptysis.**

The two commonest causes of free hæmoptysis are tuberculosis of the lungs and mitral disease—more especially mitral stenosis.

**Diabetes Mellitus.**

A weak solution of pilocarpine nitrate painted on the tongue is often useful in relieving the troublesome thirst of patients suffering from diabetes mellitus.

**Antigalactagogue.**

A 5 per cent solution of cocaine hydrochloride painted on the breast has been known to check the secretion of milk when this result was desired.

**Traumatic Neurasthenia.**

In cases of traumatic neurasthenia, provided you are satisfied that there is no real injury to the spine, the treatment resolves itself into the adoption of the Weir-Mitchell method as the only reasonable plan. It is useless simply to send such patients away for a voyage or into the country.—*Sir Victor Horsley.*

**Epithelioma and Syphilis.**

My experience has shown me the enormous number of cases where epithelioma has developed in syphilitic disease of the tongue. Hence, I go so far as to say that all chronic tongue cases should be kept under observation at regular intervals.—*Mr. Charles Ryall.*

**Muscular Atrophy.**

In progressive muscular atrophy the effect of strychnine does not admit of question. And it is incomparably more useful when injected beneath the skin than when given by the month. The nitrate is the best salt for this purpose, and may be given in a dose increasing from $\frac{1}{60}$th grain to $\frac{1}{20}$th grain once a day.—*Sir Wm. Gowers.*

**Systolic Thrill.**

A thrill is hardly ever produced in a sacculated aneurysm; and if you feel one which is, from its position, not caused by disease of the heart, it is almost certainly due to either a dilated arch of the aorta, pressure of a tumour on a vessel, or some communication between the aorta and the pulmonary artery.—*Dr. Hale White.*

**Arsenical Herpes.**

Herpes zoster may occur in a patient when he is taking arsenic, and it is not at all necessary that the doses should be large. It is a curious thing about zoster that it very rarely occurs a second time in the same patient, but there are several cases on record where people have taken arsenic and have suffered from recurring herpes.—*Sir Thos. Barlow.*

**Malignant Disease of the Larynx.**

The one symptom which is invariably present in this disease is hoarseness. This may exist many months—in some cases for a year or more—*without a single other symptom supervening.* Therefore it is the duty of every general practitioner in a case of persistent hoarseness in a person of middle age, even without any other symptom whatever being present, to make a laryngoscopic examination.—*Sir Felix Semon.*

**Blue Line on the Gums.**

An appearance very similar to the blue line produced in chronic lead poisoning may be seen in patients who use charcoal as a dentifrice.

**Sydenham on Diet.**

"More importance is to be attached to the desires and feelings of the patient, provided they are not excessive, than to doubtful and fallacious rules of medical art."

**Paroxysmal Sneezing.**

In hay fever and paroxysmal sneezing the following is a useful prescription: Quinine sulphate, gr. $1\frac{1}{2}$, arsenium iodide, gr. $\frac{1}{24}$, extract belladonna, gr. $\frac{1}{12}$. One of these pills thrice daily.—*Dr. J. B. Ball.*

**Diet in Kidney Disease.**

In some cases of parenchymatous nephritis it is a mistake to restrict the patient to a diet of milk and farinaceous food. When the patient is much debilitated and anæmic half an ounce of raw meat juice twice a day is often most beneficial, provided the pulse is not of high tension and the excretion of urea is not less than two-thirds of the normal amount.—*Dr. Cheadle.*

**The Pulse in Septic Peritonitis.**

In septic peritonitis the pulse affords much more important indications than the temperature. After abdominal operations it is more important to watch the pulse than the temperature, and the same holds good of any suspected case of peritonitis. I should feel incredulous if I heard of a case of septic peritonitis without an acceleration of the heart's action.—*Mr. C. B. Lockwood.*

**Perforated Gastric Ulcer.**

In many cases of perforated gastric ulcer the temperature as taken in the rectum is above the normal. But this, though usual, is not invariable; and it is only in the earlier hours probably that the temperature rises high; subsequently, at all events, in a good many cases it shows a tendency to fall. If there is a sub-normal temperature the case is very grave.—*Mr. A. E. Barker.*

**General Paralysis.**

Many cases of general paralysis run their whole course without any grandiose delusions, and when these do occur they rarely make themselves evident until some considerable time after a skilled observer has been able to detect many other evidences of the disease. The patients usually give no indication of having any exalted notions; on the contrary, the majority of them tell us that they are gradually losing strength.—*Dr. Risien Russell.*

**The Use of Chloral.**

Patients often come to medical men saying that one glass of alcohol produces intense flushing of the face. This symptom is not of much importance in women, but it may be in men. When you find this it is always well to ask if the person is sleeping well. Frequently you will learn that they are not, and you can then ask what they are taking to induce sleep. Chloral is often at the bottom of the complaint. The drug also produces depression and gastric disturbance.—*Dr. Maurice Craig.*

